# PI3K/mTOR dual inhibitor BEZ235 and histone deacetylase inhibitor Trichostatin A synergistically exert anti-tumor activity in breast cancer

**DOI:** 10.18632/oncotarget.14442

**Published:** 2017-01-02

**Authors:** Liyan Chen, Tiefeng Jin, Kun Zhu, Yingshi Piao, Taihao Quan, Chunji Quan, Zhenhua Lin

**Affiliations:** ^1^ Department of Pathology and Cancer Research Center, Yanbian University Medical College, Yanji 133002, China; ^2^ Department of Biochemistry and Molecular Biology, Yanbian University Medical College, Yanji 133002, China; ^3^ Department of Dermatology, University of Michigan Medical School, MI 48109-5609, USA

**Keywords:** breast cancer, BEZ235, Trichostatin A, apoptosis, autophagy

## Abstract

Molecule-targeted therapy has achieved great progress in cancer therapy. Effective drug combinations are one way to enhance the therapeutic efficacy and combat resistance. Here, we determined the effect of the PI3K/mTOR dual inhibitor BEZ235 and the histone deacetylase inhibitor Trichostatin A (TSA) on human breast cancer. We demonstrated that the combination of BEZ235 and TSA results in significant synergistic growth inhibition of multiple breast cancer cell lines. Mechanistic studies revealed that the combined therapy induced apoptosis in a caspase-dependent manner, which might be related to the further depression of the PI3K/Akt/mTOR signalling pathway. Additionally, co-treatment with BEZ235 and TSA enhanced autophagic cell death by up-regulating the expression of LC3B-II and Beclin-1. The vivo tumour modelling studies revealed that BEZ235 combined with TSA blocked tumour growth without noticeable side effects. These data suggest that the combination of BEZ235 and TSA may be a new selective strategy, which may have significant clinical application in the treatment of breast cancer patients.

## INTRODUCTION

Breast cancer is the second major cause of cancer-related death for women in world-wide [1]. The survival rates of patients with recurrences or metastases have not significantly meliorated, although numerous improvements in prevention, surgical resection and adjuvant chemoradiotherapy have caused a diminution in total mortality of breast cancer [2], underscoring the need for better strategies for both prevention and therapy.

The phosphoinositol-3-kinase/serine-threonine protein kinase B/mammalian target of rapamycin (PI3K/Akt/mTOR) signalling pathway, which plays important biological roles in normal cellular physiology, has been demonstrated to be activated in breast cancer [3–4]. Overexpression of phosphorylated Akt (p-Akt) in human breast cancer tissues using immunohistochemical techniques is found in up to 50-60% of papillary serous breast cancers [5]. Approximately 25% of breast cancer tumours have mutations in the gene encoding the kinase active p110α subunit of PI3K (PIK3CA), with the majority of mutations located in the kinase domain. There was no significant relation between PIK3CA with receptor status of HER-2 and ER/PR [6]. The expression of phosphorylated 4EBP1, a downstream effector of PI3K in breast tumour specimens, has been associated with poor prognosis [7]. These findings have encouraged the development of several different inhibitors targeting the PI3K/Akt/mTOR pathway, many of which are either in or approaching clinical trials [8–9]. BEZ235, a novel therapeutic agent that targets two molecules, including PI3K and mTOR in the PI3K/Akt/mTOR pathway [10], has demonstrated efficacy as an anti-tumour agent *in vitro* and *in vivo* in several cancers [3, 4, 11–12]. Recently, in combined treatments with melphalan, doxorubicin, and bortezomib, BEZ235 showed synergistic and additive effects on cell growth inhibition in multiple cancer cells [13–14], suggesting its potential clinical activity combined with chemotherapeutic agents.

Epigenetic modifications, including dysregulated protein acetylation, affect signalling pathways and gene expression, which accelerate drug resistance and tumourigenesis [15]. It has been demonstrated that histone deacetylase (HDAC) is overexpressed in multiple cancers, including prostate cancer, pancreatic ductal adenocarcinoma, and breast cancer, indicating that HDAC inhibitors are promising compounds for the therapy of proliferative diseases [16–17]. As the most potent reversible HDAC inhibitors, TSA is most commonly used for preclinical studies, serving as pan-HDAC inhibitors [18]. The antitumor effect of TSA may induce cell death via mitochondria dependent pathway or deregulate histone acetylation at centromeres in mitosis, causing apoptosis and abnormal chromosomal segregation [19]. According to a report, short treatment with HDAC inhibitors earlier than exposure to antitumor medicines can raise medicaments noxiousness, still in cells that are intrinsically resistant to these medicines [20], indicating that targeting multiple points of various pathways may lead to enhanced therapeutic activity.

Previous studies suggested that combining a PI3K/Akt/mTOR inhibitor and HDAC inhibitors maybe more effectual than single agents in a number of cancer cells [21–22], which represents a translatable and promising approach to cancer therapy. Our recent research also revealed that co-treatment BEZ235 with TSA exerted a synergistic inhibition on NSCLC [23]. However, a preclinical investigation of combining BEZ235 with TSA in breast cancer has not yet been reported. In this study, we investigated the joint inhibitory properties of BEZ235 and TSA in various subtypes of breast cancer cells and a xenograft model and the underlying mechanism.

## RESULTS

### Synergistic anti-tumour effect of BEZ235 and TSA

**Figure 1:**
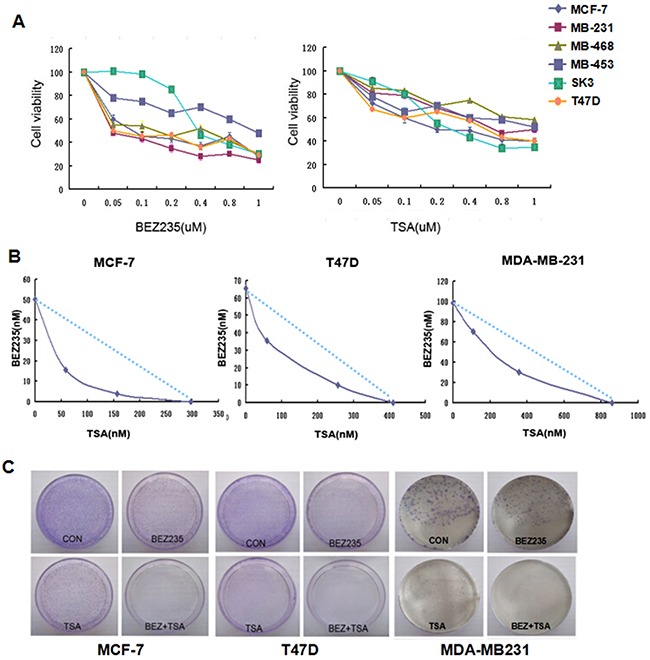
Combination treatment of BEZ235 and TSA leads to synergistic cytotoxic effect on breast cancer cells **A**. Cell viability of breast cancer cells treated by either BEZ235 or TSA for 48h was measured by MTT assay. Each cell line was analyzed in triplicate, and a representative experiment is shown. **B**. BEZ235 and TSA showed synergistically killing effect on MCF-7, T47D and MDA-MB-231 cells. **C**. BEZ235 and TSA synergistically inhibited the colony formation of MCF-7, T47D and MDA-MB-231 cells by the plate colony forming assay. Fewer colonies were formed in the group treated with both BEZ235 and TSA compared with the control group and groups treated with either BEZ235 or TSA alone.

Six breast cancer cell lines, including T47D, SK-BR-3, MCF-7, MDA-MB-231, MDA-MB-468 and MDA-MB-453, were exposed for 24, 48 or 72 hours to increasing concentrations of BEZ235 or TSA ranging from 0.1 to 1 μM, respectively. Our results showed that the proliferation abilities were significantly decreased by BEZ235 or TSA in all the above breast cancer cells (Figure 1A). The results also exhibited that MCF-7, MDA-MB-231 and T47D cells were more sensitive to BEZ235 treatment, with IC_50_ values below 0.1 μM after 48 h of incubation. In contrast, MDA-MB-468, MDA-MB-453 and SK-BR-3 with IC_50_ values ranging from 0.147 to 1.8 μM were less sensitive to BEZ235. Additionally, MDA-MB-468, MCF7 and SKBR3 cells were more sensitive to TSA treatment, with IC_50_ values below 0.5 μM, while MDA-MB-453, MDA-MB-468 and T47D cells had IC_50_ values higher than 0.5 μM (Table 1).

**Table 1:** IC_50_ value for BEZ235 and TSA as single agents in breast cancers cells

Cell lines	ER	PR	HER2	BEZ235IC50 (μM)	TSAIC50 (μM)	Combinationindex
MCF7	**+**	**+**	**-**	0.033	0.319	0.56
T47D	**+**	**+**	**+**	0.050	0.485	0.67
MDA-MB-453	**-**	**-**	**-**	1.821	1.777	1.23
MDA-MB-231	**-**	**-**	**-**	0.099	0.857	0.89
MDA-MB-468	**-**	**-**	**-**	0.147	3.331	1.89
SKBR3	**-**	**-**	**+**	0.488	0.359	1.06

Either BEZ235 or TSA can significantly inhibit the proliferation of breast cancer cells according to the MTT assay results; therefore, we further tested the combination of BEZ235 and TSA for a possible synergistic killing effect on breast cancer cells. The combination index (CI) was used to decide whether the combined treatment of drugs is synergistic, additive, or antagonistic by the Chou and Talalay method [24]. As represented in Table 1 and Figure 1B, the results indicated that rather than simple additive killing, the combination of BEZ235 and TSA exerted a highly synergistic cytotoxic effect on MCF-7 cells with a CI value below 0.7. Similarly, a synergistic effect of BEZ235 plus TSA was also observed in T47D cells and MDA-MB-231, with no correlation with ER, PR and HER2 status.

To further confirm the potential inhibitory property of co-treatment with BEZ235 and TSA on breast cancer cells, we aimed to ascertain if the combination might inhibit colony formation of breast cancer cells. After 14 days of growth in the combination drug group, fewer and smaller colonies were counted compared with either the control or single drug groups in MCF-7, T47D and MDA-MB-231 cells (Figure 1C). These results, along with the viability data, confirmed the synergistic antitumor effect of BEZ235 and TSA combination on these breast cancer cells.

### Genome-wide analysis of BEZ235 and/or TSA induced differential gene expressions in breast cancer cells by gene chip

**Figure 2:**
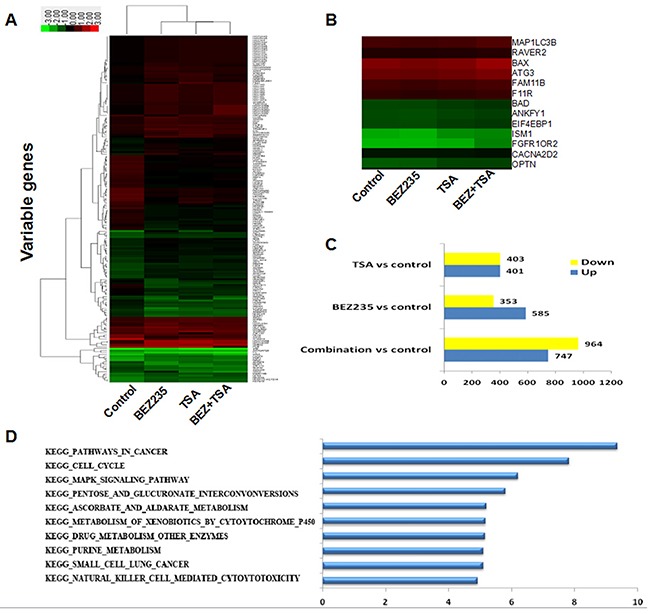
Genome-wide analysis of BEZ235 and/or TSA induced genes in MCF-7 cells **A**. Unsupervised hierarchical clustering using the selected genes. **B**. Visualization of genes with their expression profiles. **C**. Differencially expressed genes from each treatment compared to the control in MCF-7 cells. **D**. Pathway enrichment analyses of MCF-7 treated with BEZ235 and/or TSA. Data were z-score normalized.

To estimate the potential interaction of PI3K/mTOR signalling pathways and HDAC inhibition in a more comprehensive manner, a transcriptome analysis was performed utilizing microarrays after 24 hours of treatment with BEZ235 and/or TSA in MCF-7 cells. After filtering and normalization, gene expression alteration in samples was calculated. Microarrays assess concurrent alterations in transcription levels on an individual basis, leading to a long list of genes which have changed transcript levels significantly compared to control cells. Here, we found 747 up-regulated and 964 down-regulated genes (fold change ≥2.0) in the combination treatment group of MCF-7 cells, and the numbers of genes induced was much larger than that induced by BEZ235 (585 up-regulated and 353 down-regulated) or TSA (401 up-regulated and 403 down-regulated) (Figure 2).

These alterations in gene expression, however, do not exist as independent actions within the cell but are dominated in a co-ordinated manner and always interconnected. Pathway Analysis is an impartial approach to determine whether genes and the proteins differentially expressed are enriched in some particular pathways, providing insight into the biological significance of the changes that are observed. According to all pathways in the genetic information (KEGG, BIOCARTA), pathway enrichment analysis of differential genes and hierarchical cluster analysis were performed. Importantly, in sorting differentially expressed genes pursuant to their differential expression level, our data clearly revealed that these focus genes have functions in cancers, cell death and survival, cellular development, and development disorders. The combination group cells had significantly higher expression levels of genes associated with apoptosis and autophagy such as *BAX* (encoding bax), *CASP9* (encoding Caspase 9), *MAP1LC3B* (encoding LC3B) and but lower expression levels of genes, such as *BCL-2* (encoding Bcl-2) than the other group.

### Effect of BEZ235 and TSA treatment on PI3K/mTOR signalling

**Figure 3:**
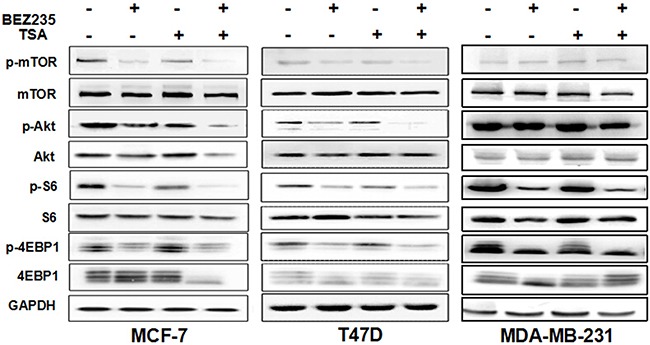
Effect of BEZ235 and TSA co-treatment on PI3K/mTOR Signaling Western blot showed increased inhibition of the PI3K/mTOR pathway in breast cancer cells MCF-7, T47D and MDA-MB-231 co-treated with BEZ235 and TSA for 48h. Each protein was analyzed in triplicate, and a representative experiment is shown.

To elucidate the signalling pathway inhibition of the combination of TSA and BEZ235 in breast cancer cells, PI3K/Akt/mTOR pathway molecules were detected after 48 h of drug treatment. As shown in Figure 3, the PI3K/mTOR inhibitor BEZ235 reduced the phosphorylation of S6 (S240/244), Akt (S473), mTOR (S2448), and 4EBP1 (S65) in the cultured MCF-7, T47D and MDA-MB-231 cells. Although TSA treatment alone did not significantly affect PI3K/AKT/mTOR pathway protein phosphorylation, if in combination with BEZ235, it further inhibited the phosphorylation of S6(S240/244), Akt (S473) and 4EBP1 (S65) in various degree in breast cancer cells relative to single treatments.

### Co-treatment with BEZ235 and TSA synergistically induce apoptosis signalling

To determine if the synergistic growth inhibition of BEZ235 and TSA results from apoptosis, we used flow cytometry, Hoechst 33342 staining, and western blot to observe nuclear fragmentation and apoptosis related protein expression in T47D, MCF-7 and MDA-MB-231 cells treated with BEZ235 and/or TSA.

**Figure 4:**
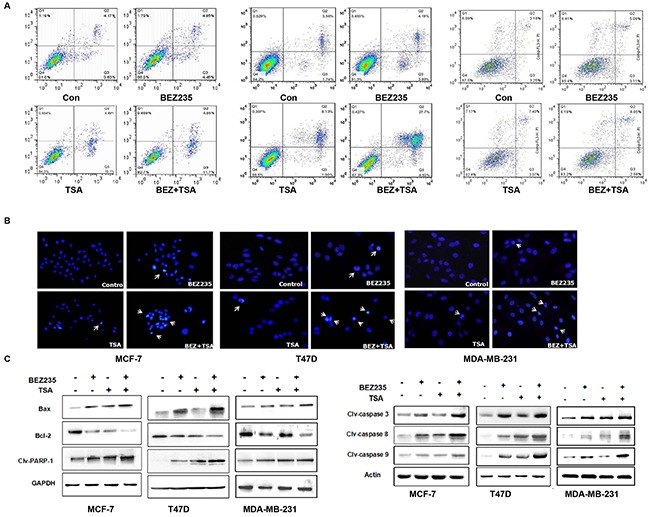
Co-treatment with BEZ235 and TSA synergistically induced apoptosis signaling **A**. Flow cytometric analysis of Annexin V-FITC/PI stained of breast cells after treatment with BEZ235 and TSA alone or combination. **B**. BEZ235 and TSA co-treatment exacerbated the apoptosis of breast cancer cells by hoechst 33342 staining. **C**. Apoptosis induced by BEZ235 and TSA co-treatment in breast cancer cells by western blotting. Bax proteins was greatly up-regulated, and Bcl-2 proteins significantly decreased in combination group. Cleaved caspase-3, -8 and -9 proteins and PARP-1 were significantly higher in combination group than those in either single group or control group. Statistic analysis is presented from the results of three independent western blot assays.

As shown in Figure 4A, co-treatment with BEZ235 and TSA induced more apoptosis of breast cancer cells compared to treatment with either agent alone (p <0.05). The apoptotic rate in T47D cells treated with BEZ235 and TSA was 33.23%, and it was significantly higher than that in the BEZ235 group (8.18%) and TSA group (10.03%) after treatment for 48 h. Similarly, in MCF-7 and MDA-MB-231 cells, co-treatment of BEZ235 and TSA resulted in an increased apoptosis rate compared with other groups.

The Hoechst 33342 staining results revealed that the apoptotic cells were more frequently observed in breast cancer cells treated with the BEZ235 and TSA combination compared with the groups treated with BEZ235 or TSA alone (Figure 4B). Additionally, the cells possessed marked morphological changes in the combination group than either the BEZ235 or TSA group. The cells of the control group showed round and homogeneous nuclei; however, following co-treatment with BEZ235 and TSA for 48 h, alterations in the size, shape, and structure of the cell nuclei were observed as well as the condensed chromatin and cell shrinkage.

Moreover, western blot data showed that the expression level of Bcl-2 was down-regulated following BEZ235 and TSA co-treatment, and Bax expression was up-regulated under the same conditions (Figure 4C). Importantly, the ratio of Bcl-2/Bax was significantly decreased, which suggests that the Bcl-2/Bax plays an important role in breast cancer cell progression regulated by the BEZ235 and TSA combination. Our data also showed that cleaved caspase-3, caspase-8, caspase-9 and poly ADP ribose polymerase-1 (PARP-1) were all increased in breast cancer cells. These data suggest that cell apoptosis induced by dual drug treatment was caspase-dependent, indicating that the combination of BEZ235 and TSA exerts an anti-breast cancer effect through not only the mitochondrial pathway but also the death receptor pathway.

### Autophagy inhibitor 3-MA increases the apoptotic cell death induced by combination with BEZ235 and TSA

**Figure 5:**
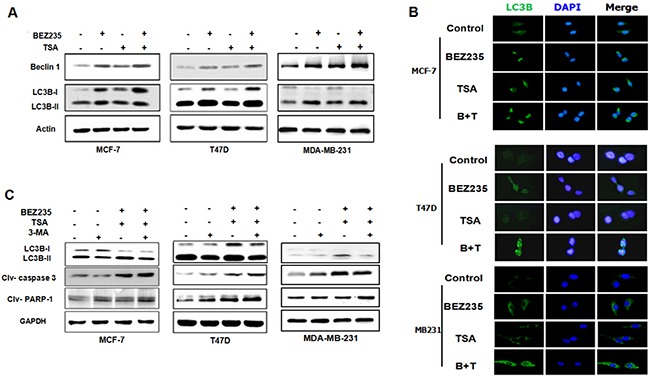
Co-treatment with BEZ235 and TSA enhanced autophagy in breast cancer cells **A**. MCF-7, T47D and MDA-MB-231 cells were treated with the indicated concentration of BEZ235 and/or TSA for 48 h. Then, the total cell lysates were prepared and western blot analyses were performed for autophy related protein (Beclin-1, LC3B). **B**. LC3B expression in breast cancer cells were visualized by immunoreflurensence. The combination group show greater punctuate pattern of LC3B staining, typical of autophagy compared with single drug group. **C**. 3-MA reduced the autophagy induced by co-treatment with BEZ235 and TSA, while enhanced the apoptosis in breast cancer cells.

Given the critical role of mTOR in negatively regulating autophagy [25], we then studied whether co-treatment with BEZ235 and TSA induce autophagy in breast cancer cells. Using western blot, we detected increased levels of LC3B-II, a lysosome-bound form of LC3B, in MCF-7, MDA-MB-231 and T47D cells exposed to BEZ235 and TSA (Figure 5A). Beclin1 expression was also significantly increased at the same time, indicating the autophagy induced by the dual drug treatment in breast cancer cells. Moreover, in agreement with western blot data, we observed enhanced punctate staining of FITC-LC3B in cells when exposed to BEZ235 and TSA co-treatment (Figure 5B), compared with either single drug group or the control group, suggesting the formation of autophagosomes. Collectively, these results demonstrate that the combination of BEZ235 and TSA exerted anti-breast cancer effects through inducing not only apoptosis but also autophagy.

To explore the interaction between autophagy and apoptosis in more detail, we detected the immunoblotting of LC3B, caspase-3 and PARP-1 of breast cancer cells with 3-MA pre-treatment before combined BEZ235 and TSA treatment, an inhibitor of autophagy. Here, we found that with the addition of 3-MA, the expression of LC3B-II in the dual drug group was lower than in the absence of 3-MA, indicating that autophagy induced by the combination of BEZ235 and TSA was blocked by 3-MA. More importantly, we found that the apoptotic executioners, cleaved caspase-3 and PARP-1, increased significantly in the presence of 3-MA (Figure 5C). Collectively, these observations suggested that as a parallel response to the BEZ235 and TSA combination strategy, autophagy accelerated the cell death of breast cancer cells in addition to apoptosis.

### Combination therapy in breast cancer xenograft model

**Figure 6:**
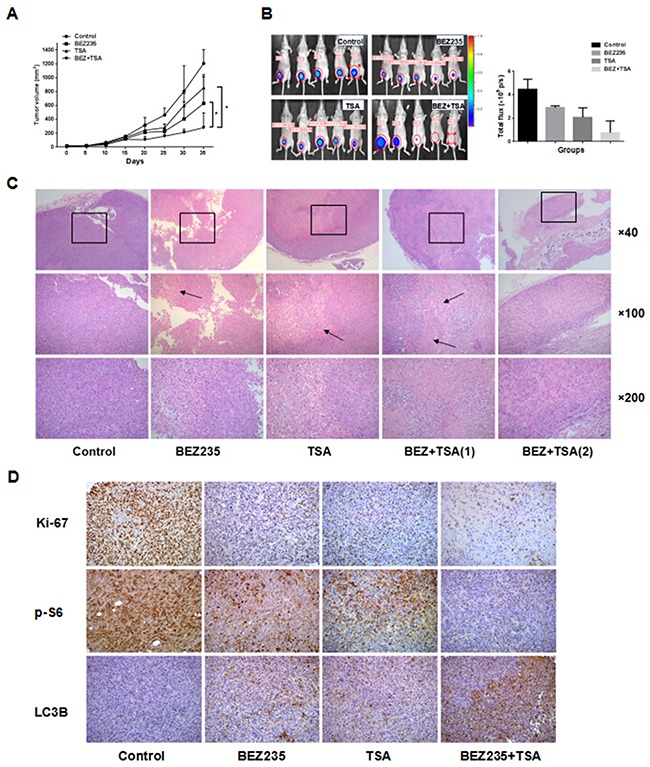
*In vivo* treatments of MDA-MB-231 xenografts **A**. Mice bearing MDA-MB231 tumors were treated every other day with vehicle control (5% DMSO, i.p.), BEZ235 (30 mg/kg/d, p.o.), TSA (1.5 mg/kg/d, i.p.), or the combination (BEZ235+TSA) for 35 days. Measurements are displayed as mean±SE. *P<0.05, **B**. Optical imaging of mice bearing MDA-MB-231 tumor xenografts. Mice (n=5) were anesthetized and scanned at day 35. The MDA-MB-231 tumor graft is indicated by a cycle. **C**. HE staining of sections from xenografts tissues under a microscope. Necrosis and lymphocytes infiltrating were more frequently observed in the xenograft tissues of co-treatment group compared with either BEZ235 or TSA group, and the DMSO treated xenografts showed focal necrosis foci (arrow) and lymphocytes infiltration. **D**. Immunohistochemical staining for Ki67, p-S6 and LC3B protein in tumour specimens from xenografts.

To better recapitulate the clinical setting, we tested the effect of treatment with BEZ235 and/or TSA on the growth of established tumour xenografts of MDA-MB-231 cells implanted in the mammary gland pad of nude mice. As shown in Figure 6, treatment with BEZ235 or TSA alone significantly reduced tumour growth, resulting in lower mean tumour volume of the tumours, compared to control treatment (vehicle alone). Notably, combined treatment with BEZ235 and TSA completely abrogated the growth of tumour xenografts, an effect significantly superior to treatment with BEZ235 or TSA as a single agent. After approximately 35 days of treatment, neither a single agent nor the combination of BEZ235 and TSA caused weight loss or other toxicity to the nude mice at the doses that were used in both models. Moreover, we confirmed that the effect of treatment with these agents together was associated with marked tumour necrosis by H&E staining. IHC staining also showed lower expression of Ki-67 and p-S6 and higher expression of LC3B in the co-treatment group than in the other groups, which was consistent with the *in vitro* findings.

## DISCUSSION

The understanding of breast cancer potential biology has risen exponentially recently, and improvements in molecular biology have unveiled a substantial sum of genomic aberrations. Recent researches unveiled that many aberrations centered on certain key pathways of signal transduction in cancer cell, including PI3K/Akt/mTOR and epigenetic alterations. Future strategies in consequence are likely to utilize combined two or more targeted drugs to further decelerate the traffic of signals pathways, therefore, achieve optimum and undergone clinical benefit.

Recent studies indicate that combined PI3K/mTOR inhibitor with HDAC inhibitor may be more efficacious than single drug in some cancer cells [25–26]. Erlich *et al*. reported that the PI3K/mTOR inhibitor BGT226 (Novartis) and BEZ235, in combination with the HDAC inhibitor panobinostat, inhibit the viability of head and neck squamous carcinoma cells better than single drug [27]. The works of Kathleen *et al*. illustrated that HDAC inhibitor TSA and the mTOR inhibitor MLN0128 synergistically inhibited the proliferation and induce apoptosis of breast cancer cell lines [28]. In the present study, we found that BEZ235 and TSA, if administered alone, exert antitumour activity against breast cancer cells in *vitro*. These findings are consistent with previous reports that have documented the preclinical single agent activity of BEZ235 or TSA against lung cancer and PDAC cells [23, 29–30]. More importantly, for the first time, we reported that co-treatment with BEZ235 and TSA could selectively synergistically affect the viability of MCF-7, MDA-MB-231 and T47D cell lines compared with other breast cancer subtypes, suggesting that different breast cancer cell lines have varied sensitivities to the combination treatment. Additionally, the dual drug treatment is superior to single-treatment *in vivo*, which is consistent with the results of the *in vivo* experiments.

In some breast cancers, the PI3K/Akt/mTOR pathway is overactive irrelevant to HER2 and ER/PR status. Akt, a serine/threonine-specific protein kinase, stimulates mTOR, resulting in protein synthesis increasing via its effectors 4EBP1 and S6K [31–32], promoting polysome formation and translation of transcription factors modulating tumour cell growth. Akt also phosphorylates BAD, BIM and caspase, which leads to inhibition of apoptosis [33]. Thus, Akt is known as a key point bonding cell growth, cellular metabolism and cell apoptosis. In this study, we found that co-treatment with BEZ235 and TSA caused a marked reduction in p-Akt (Ser473) levels, abolishing the feedback activation of PI3K-Akt due to p-mTOR (Ser2448) inhibition, in addition to maintaining marked attenuation of p-S6 (Ser240/244) and p-4EBP1 (Ser65) levels caused by depletion of p-mTOR (Ser2448) activity. Phosphorylation of 4EBP1 appears to be a key regulator of the proliferation and survival of breast cancer cells. Consequently, a marked depletion of p-4EBP1 (Ser65) levels upon combined treatment with BEZ235 and TSA would inhibit the growth and survival of breast cancer cells. Therefore, the synergistic effect on breast cancer cells is connected with further inhibition of the PI3K/mTOR signalling pathway.

For most drugs inducing cancer cell death, cell apoptosis is a common mechanism. Venkannagari *et al*. reported that BEZ235 and Panobinostat (HDAC inhibitor) can synergistically suppress pancreatic cancers through the TORC1/4EBP1 signalling pathway and induce apoptosis [34]. Here, using a FACS assay and Hoechst 33342 staining, we found that co-treatment with BEZ235 and TSA could synergistically induce apoptosis compared with either BEZ or TSA alone. It is well known that apoptosis is regulated by various signalling pathways, including the death ligand pathway and the mitochondrial pathway. In the process of the mitochondrial pathway, Bcl-2, the anti-apoptotic protein and Bax, the pro-apoptotic protein were actived, which activate caspase-3 and caspase-9 and accelerate the secretion of cytochrome c in the downstream [35–36]. Once activated, caspase-3 cleaves PARP-1 into two fragments, promoting DNA fragmentation and triggering apoptosis, indicating that an impaired DNA repair mechanism is associated with the induction of apoptosis [37–38]. In another signal pathway of apoptosis, the death receptor pathway leads to the activation of a caspase cascade involving caspase-8 and caspase-3. As initiator caspase, caspase-8 produce a cascade reaction in response to extracellular signals from apoptosis-inducing ligands [39]. In this study, our results showed that co-treatment of BEZ235 and TSA resulted in greatly reduced Blc-2 protein expression in breast cancer cells, whereas Bax protein increased, in agreement with previous reports, which suggests that the ratio of Bcl-2/Bax is significant in the survival of drug-induced apoptosis in cancer cells, rather than Bcl-2 alone [40–42]. Moreover, cleaved caspase-3, 8, and 9 proteins were all significantly increased, indicating that the caspase-dependent pathway was involved in the BEZ235 and TSA co-treatment. The mitochondrial pathway and the death receptor pathway maybe the mechanisms that the combination treatment inhibits growth and induces apoptosis in breast cancer cells.

Recent studies demonstrated the association between autophagy and cancer. Concurrent induction of apoptosis and autophagy in some cancer cells was reported for several anticancer compounds, such as cannabidiol in breast cancer MDA-MB-231 cells, which provides a mechanism that cancer cells may survive stresses imposed by therapy [43]. So far over 35 proteins are considered to be essential in autophagy occurrence and progression containing Beclin1 and LC3B [44]. The conversion of LC3B to LC3B-II, the lower migrating form in autophagosomes have been considered as indicator of autophagy [45]. Chen *et al* revealed that there were 9 cell lines that had allelic Beclin-1 deletions among 22 breast cancer cell lines by a FISH analysis [46]. High frequency of monoallelic *BECN1* gene deletion has been found in up to 75% cases of human ovarian, breast and prostate tumours. Thus, Beclin-1 is considered a tumour suppressor gene. The novel finding in this study is that co-treatment with BEZ235 and TSA potently induces autophagy in breast cancer cells while inhibiting cell growth and initiating apoptosis, evidenced by the detection of increased protein levels of LC3B-II and Beclin1 and punctate staining of FITC-LC3B bound in autophagosomes. These results suggest that co-treatment with BEZ235 and TSA may lead to the engagement of autophagic cell death and tumour cell growth inhibition.

There is a close relationship between apoptosis and autophagy during cell death. The intricate interaction between these mechanisms is a substantial challenge in cancer therapy. Under some conditions, autophagy and apoptosis can exert synergetic effects, while in other situations, autophagy can be initiated only if apoptosis is repressed [47]. 3-MA, an inhibitor of the autophagy, has been uncovered to block the formation of preautophagosome, autophagosome, and autophagic vacuoles [48]. As expected, we found that 3-MA diminished the increase in the autophagic protein LC3B-II, induced by the combination group, which is consistent with previous reports. Additionally, we found that the cleavage of caspase-3 and PARP-1 were also improved in varying degrees by 3-MA in breast cancer cells treated by the BEZ235 and TSA combination group, indicating that dual drug treatment may trigger apoptosis and autophagy, leading to cell death of breast cancer cells via a caspase-dependent pathway.

In summary, our findings demonstrate that the simultaneous suppression of the combination of the PI3K/mTOR inhibitor BEZ235 and the pan-HDAC inhibitor TSA is more effective than single agent in inhibiting the viability of breast cancer cells *in vitro* and tumour progression *in vivo*. This combination is also effective in inducing apoptosis and enhancing autophagy in tumour regression of these breast cancer cells, which could be a new selective strategy for breast cancer patients.

## MATERIALS AND METHODS

### Cell culture

Human breast cancer cells MCF-7, T47D, MDA-MB-231, MDA-MB-453, MDA-MB-468 and SK-BR3 were obtained from American Type Culture Collection (ATCC, Manassas, VA). The cell lines were cultured in DMEM and PRMI1640 medium supplemented with 10% fetal bovine serum (FBS) and 1% penicillin/streptomycin at 37°, 5% CO_2_. When the cells were spread the bottle bottom of 80%, transferred of culture by 0.25% trypsin digestion every 2~3 days. Cells were thawed and cultured for 3~5 passages, then frozen in aliquots in -80° and/or liquid nitrogen until use.

### Chemicals and antibodies

BEZ235, TSA and 3-MA were purchased from Selleck company (Selleck, Shanghai, CHINA). Antibodies for western blotting and flow cytometry were: mouse anti-cleaved caspase-3, mouse anti-cleaved caspase-8, mouse anti-cleaved caspase-9, mouse anti-p-Akt, mouse anti-Beclin-1, mouse anti-LC3, mouse anti-S6, mouse anti-p-S6, mouse anti-4EBP1, mouse anti-p-4EBP1, mouse anti-mTOR (Cell Signaling, USA), mouse anti-PARP-1 (Abcam, USA).

### Cell viability assay and synergy calculations

Cell viability was measured using the MTT [3-(4,5-Dimethylthiazol-2-yl)-2,5-diphenyl tetrazolium] dye reduction method. Cells in DMEM or PRMI1640 medium with 10% FBS were plated into 96-well plates (2×10^3^ cells/100μL/well) and cultured with indicated compounds for 24 h, 48 h, 72 h. The absorbance value (OD) of the wells was measured with a microplate reader at test and reference wavelengths of 570 nm. Percent growth was reported relative to untreated controls. Each experiment contained at least triplicate samples and was performed at least three times. The combination index (CI) for each drug combination was obtained using the commercially available software Calcusyn (Biosoft, Ferguson, MO). CI<1, CI=1, and CI>1 represent synergistic, additive or antagonistic interaction of the two agents, respectively.

### Flow cytometry

Cells were harvested by 0.25% trypsin, then collected by centrifugation at 1000×g for 5 min at RT. After prepared cell solution at a concentration of 1× 10^6^ cells/mL, cells were mixed in Binding buffer, and incubated with 5uL of Annexin V-FITC and 5uL Propidium iodide (PI) (1:100, FITC Annexin V Apoptosis Detection kit II, BD Bioscience, USA) for 20 min in darkness. Fluorescence was measured by FACS caliber cytometer (BD Company, USA) using standard software.

### Colony formation assay

Prepared the concentration of 100 cells/mL cells and plated 1 mL of cell solution to 6 cm dish to expect plating 100 cell in each dish. After adding the drug to the medium at indicated concentration for 48 h, medium was replaced and colonies allowed to form until clearly visible, usually an additional 14 days. Cells were fixed with 4% paraformaldehyde for 1 h at RT, then stained with Giemsa. The cloning efficiency was calculated by dividing the number of wells containing proliferating cells with the total number of cell-plated wells.

### Immunofluorescence staining (IF)

Cells were grown on cover slips to 60~70% of cell confluence, then fixed by methanol for 30 min and permeabilized with 0.5% Triton X-100 for 5 min. After washing in PBS, the fixed cells was incubated in 3% albumin bovine serum (BSA) for 1 h at RT. Cells were incubated with primary antibody (1:50) for 1 h and the secondary antibody (against Alexa Fluor^®^488 goat anti-rabbit IgG and Alexa Fluor^®^568 goat anti-mouse IgG, 1:1000) for 30 min at RT. Staining cells with 0.1~1 mg/mL 49-6-diainidino-2-phenylindole (DAPI) for 5 min, cells were observed with Leica confocal microscope TCS SP5 II.

### Western blot analysis

Whole cells lysate were prepared and electrophoretic sepearated by sodium dodecyl sulfate-polyacrilamide (SDS-PAGE) gel. Then polyvinylidene fluoride (PVDF) membrane electrotransferred was incubated with 5% skim milk solution for 1 h at RT, and overnight at 4°C with primary antibodies (1:1000 ~ 1:5000). After washing the membrane for three times with PBS-T, PVDF membrane was shaked with secondary antibody at RT for 1 h. Then protein-antibody complexes were detected by enzyme-linked chemiluminescence (ECL) kit (ECL, Pierce Biotechnology). Results were analyzed quantitatively using Chemiluminescent and Fluorescent Imaging System (Sage, champchemi professional, China).

### Hoechst staining

Treated cells were fixed with methanol acetic acid for 10 min followed by staining with Hoechst 33342 at 1 mg/mL staining at room temperature at dark for 5 min. Cells were washed twice with PBS, examined and immediately photographed under a fluorescence microscope.

### Animal studies

Animal experiments were approved by the animal ethics committee of the Yanbian university, China and performed in accordance with the regulations of the Service of Consumables and Veterinary Affairs-Division of Animal Protection (SCAV-EXPANIM). Female nude mice aged 4~6 weeks were obtained from Beijing Vital River Laboratories (VRL), China, and housed in pathogen-free conditions and maintained at 25±1° under a natural light-dark cycle in a well-ventilated room. 5×10^6^ MDA-MB-231 cells were injected into the mammary fat pad of nude mice. When tumor reached to 200 mm^3^, mice were randomized into four groups (n=5, in each group): DMSO, BEZ235(30mg/kg/d, p.o.), TSA(1.5mg/kg/d, i.p.), and BEZ235+TSA groups. Animals were sacrificed after 35 days of treatment, and remove the tumor in formalin, part of the organization after cut up fresh frozen in liquid nitrogen for further analysis.

### Statistical analysis

Statistical analyses were conducted using SPSS 17.0 software package (SPSS Inc., Chicago, IL, USA). Data from experiments were expressed as means±standard deviation (SD), and evaluated by analysis on factorial design of two factors and one-way ANOVA. Differences were considered statistically significant at *p* < 0.05.
